# New Studies of Pathogenesis of Rheumatoid Arthritis with Collagen-Induced and Collagen Antibody-Induced Arthritis Models: New Insight Involving Bacteria Flora

**DOI:** 10.1155/2021/7385106

**Published:** 2021-03-25

**Authors:** Ryoichi Hashida, Yasunori Shimozuru, Jessica Chang, Ibis Agosto-Marlin, Takaki Waritani, Kuniaki Terato

**Affiliations:** Chondrex Inc., 16928 Woodinville-Redmond Rd NE STE B101, Woodinville, WA 98072, USA

## Abstract

Much public research suggests that autoimmune diseases such as rheumatoid arthritis (RA) are induced by aberrant “self” immune responses attacking autologous tissues and organ components. However, recent studies have reported that autoimmune diseases may be triggered by dysbiotic composition changes of the intestinal bacteria and an imbalance between these bacteria and intestinal immune systems. However, there are a few solid concepts or methods to study the putative involvement and relationship of these inner environmental factors in RA pathogenesis. Fortunately, Collagen-Induced Arthritis (CIA) and Collagen Antibody-Induced Arthritis (CAIA) models have been widely used as animal models for studying the pathogenesis of RA. In addition to RA, these models can be extensively used as animal models for studying complicated hypotheses in many diseases. In this review, we introduce some basic information about the CIA and CAIA models as well as how to apply these models effectively to investigate relationships between the pathogenesis of autoimmune diseases, especially RA, and the dysbiosis of intestinal bacterial flora.

## 1. Introduction

Autoimmune diseases such as rheumatoid arthritis (RA) have been believed to be caused by an overactivated immune system recognizing and attacking “self” tissues as “foreign” tissues. Unfortunately, triggers of this immune activation have not yet been elucidated.

RA susceptibility is linked to the major histocompatibility complex (MHC) class II genes [1–3], and the severity of RA in patients who have the HLA-DR4 gene tends to be high. However, RA is not a disease specifically induced in only HLA-DR4 positive individuals; rather, it may depend on conditions in hosts and environmental factors including lifestyle habits (smoking) [4], diet [5], periodontal diseases [6–8], gastrointestinal diseases [9], mental stress [10], dysbiosis of intestinal bacteria [11, 12], and immunological dysfunctions correlated with aging [13].

Recent advances in 16S ribosomal RNA analysis of fecal bacteria show dysbiosis, an abnormal balance of intestinal bacteria, is a common factor in many autoimmune diseases including RA [14–18]. Other common factors may include changes in substances produced by intestinal bacteria and destruction of mucosal barriers and gastrointestinal defense mechanisms. In addition, it is also important to assess the contribution of antibody reactions, which play important roles in immune defense [19, 20].

Collagen-Induced Arthritis (CIA) and Collagen Antibody-Induced Arthritis (CAIA) are useful animal models of RA and have been used for the functional analysis of pathological genes and for evaluating the anti-inflammatory and anti-RA drugs. Based on significant accumulated information, both models can be useful to investigate the contribution of conditions in hosts and environmental factors in autoimmune diseases. In this review, we introduce some basic information about CIA and CAIA, and new approaches as well as strategies for using these models to understand better the contribution of bacteria in the pathogenesis of RA.

## 2. CIA and CAIA as Animal Models of RA

### 2.1. Development of CIA

#### 2.1.1. Induction of CIA

Arthritis animal models such as adjuvant arthritis, CIA, and CAIA are convenient for RA research because the severity of inflammation is easily evaluated and quantitatively analyzed. The widely used CIA model was developed by Trentham et al. and consists of immunizing Wistar rats with type II collagen, who subsequently develop polyarthritis in the paws [21]. After their discovery, chronic and progressive arthritis models were induced in rats [22, 23] and mice [24] as well as in some primates (squirrel monkeys [25], rhesus [26], cynomolgus [27], and marmosets [28]) by immunization with type II collagen emulsified in Complete Freund's Adjuvant (CFA). These models are now widely used and considered standard RA models in preclinical studies.

#### 2.1.2. Specificity of Serum Antibody: Nonimmunogenicity of Autologous Type II

**Figure 1:**
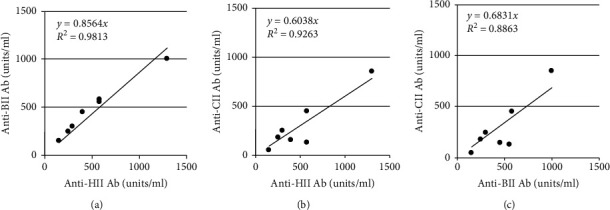
Specificity of anti-type II collagen antibodies in RA patients. Cross-reactivity of autoantibodies (Ab), purified from RA patient serum using an HII-affinity column: bovine type II collagen (BII), chick type II collagen (CII), and human type II collagen (HII). (a) HII versus BII. (b) HII versus CII. (c) HII versus CII.

The CIA models have similar immunological and pathological characteristics to RA. CIA can be induced in several mouse strains expressing susceptible major histocompatibility complex (MHC) genes. CIA susceptibilities in mice depend on the MHC H-2 haplotype [29], similar to that in humans where RA severities depend on the MHC class II molecule, HLA-DR [1–3]. Antibodies in the serum from RA patients react to their autoantigen, human type II collagen (HII), and heterologous chicken and bovine type II collagen (CII and BII) as well, because they recognize a common epitope in type II collagen (Figure 1). These cross-reactivities were observed in CIA models in which serum antibodies cross-reacted to immunized CII or BII and their own rat or mouse type II collagen (MsII) [30, 31].

This evidence suggests that RA patients may be sensitized to heterologous type II collagen similar to that in CIA animals. Of note, serum from RA patients does not contain HII-specific antibodies [31], and the immunization of mice with MsII did not induce arthritis because the MsII is not recognized as an antigen by their immune systems [32]. This suggests that autologous type II collagen is not an antigen that induces antibody production.

CIA is not a direct RA animal model because of its different disease mechanisms. For example, the concentration of serum anti-type II collagen antibodies in arthritic CIA mice is significantly higher than in RA patients. DBA/1 mice develop CIA within 6–8 weeks after immunization, and the serum anti-type II collagen antibodyconcentration can reach mg/ml levels. In contrast, the serum anti-type II antibody concentration in RA patient is in *μ*g/ml levels. RA patients harboring the HLA-DR4 gene with severe arthritic inflammation have low levels of anti-type II collagen autoantibodies, in contrast to CIA-susceptible DBA/1 mice. Therefore, RA can be characterized into two types, both of which are related to environmental factors, but differ regarding how antibodies induce pathology [19, 20].

#### 2.1.3. Induction of Arthritis by Serum Antibody Transfer

CIA can be induced by the transfer of serum from arthritic CIA mice into naïve mice [33]. Furthermore, the injection of a serum IgG fraction containing anti-type II collagen antibodies from RA patients also induced arthritis in naïve mice [34]. This evidence suggests autoantibodies against autologous type II collagen are essential for the induction of CIA.

#### 2.1.4. Contribution of T Cells in the Induction of CIA

Type II collagen is a T cell dependent antigen and researchers have suggested that CIA and RA are induced by T cell activation. A study reported that the transfer of T cell clones established by repeated type II collagen stimulation induced arthritis in mice [35]. However, the T cell clones were actually specific to pepsin which was used in the extraction of collagen, and this result misled the researchers into believing that T cells were reactive to autologous MsII [36]. Finally, a study, which used pepsin-free CB-peptides of MsII obtained by cyanogen bromide (CB) digestion, confirmed that mouse T cells only recognized species-specific epitopes of immunized type II collagen and did not react to common epitopes of heterogeneous type II collagen and MsII (unpublished data). However, these findings cannot conclude that T cells independently involve progression and aggravation of arthritis. In arthritis, T cells differentiate into various subtypes and release inflammatory and anti-inflammatory cytokines and chemokines, all of which contribute to the augmentation of inflammation and degradation of connective tissues. We found that irradiation of 2 Gy X-rays in CIA mice induced more severe inflammation in the areas of the tails where the adjuvant was injected, indicating the importance of T cells in inflammatory conditions (unpublished data).

### 2.2. Development of CAIA

#### 2.2.1. Arthritogenic Epitopes on Type II Collagen in CIA and RA

**Figure 2:**
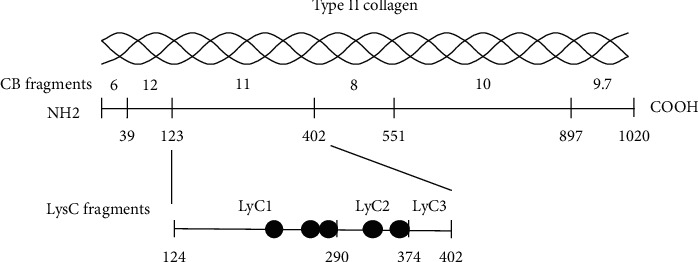
Localization of arthritogenic epitopes on cyanogen bromide (CB) digested type II collagen fragments (CB 6, 8, 9.7, 10, 11, and 12). The circles represent the epitopes of the arthritogenic monoclonal antibodies on endoproteinase (LysC)-digested CB11 fragments (LysC1, LysC2, and LysC3).

The restricted location of arthritogenic epitopes recognized by autoantibodies in the sera from CIA mice and RA patients was studied using CB-peptides of type II collagen. When DBA/1 mice were immunized with CII or BII, arthritogenic epitopes were found only in CB11 (Figure 2), the eleventh fragment by CB cleavage [37], and when B10.RIII mice were immunized with BII, arthritogenic epitopes were found in CB8 [38]. In CIA-susceptible Wistar rats, the autoantibodies recognized CB11. In contrast, in monkeys [39] and humans [40], arthritogenic epitopes were widely distributed in CB7 through CB11 depending on the individual MHC backgrounds.

#### 2.2.2. Induction of Mouse Arthritis with a Cocktail of Monoclonal Antibodies

**Figure 3:**
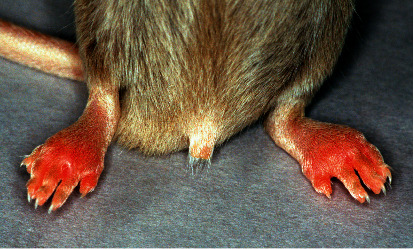
Arthritis induced by the IV injection of an anti-type II collagen monoclonal antibody cocktail. Arthritis in DBA/1 mice on day 8 after the IV injection of the four-clone monoclonal antibody cocktail (10 mg), which recognizes the multispecies common epitope on the CB11 fragment of type II collagen. Mice developed polyarthritis in the paws.

The distribution of CB11 arthritogenic epitopes in DBA/1 mice was demonstrated by the immunization of mice with the renatured CB11 fragment of type II collagen [37]. Based on this discovery, we succeeded in inducing arthritis, called Collagen Antibody-Induced Arthritis (CAIA), in normal mice by the intravenous (IV) administration of an antibody cocktail containing four monoclonal antibodies that recognized the CB11 arthritogenic epitopes (Figure 3) [41].

Moreover, by adding another monoclonal antibody into the four-clone antibody cocktail, we established a more effective arthritis model [42]. Three of the clones, A2-10 (Ig G2a), D1-2G (IgG2b), and D2-112 (IgG2b), recognize the LysC1 (124–129) fragment consisting of 167 amino acids obtained by digesting CB11 with LysC, an endoproteinase, and the two other clones, F10-21 (IgG2a) and D8-6 (IgG2a) recognize the LysC2 (291–374) fragment (Figure 2). Due to the ability of the monoclonal antibody injection to bypass antibody production steps, the CAIA model has limitations for studies on antigen recognition by antigen-presenting cells and T cells. This was confirmed in CAIA induction using scid/scid mice who lack T cells [43]. However, CAIA can be used as a negative control with CIA studies to study the mechanism of these antigen recognition and antibody production steps.

#### 2.2.3. Synergistic Effects of Type II Collagen Antibodies and Bacterial Toxins

**Figure 4:**
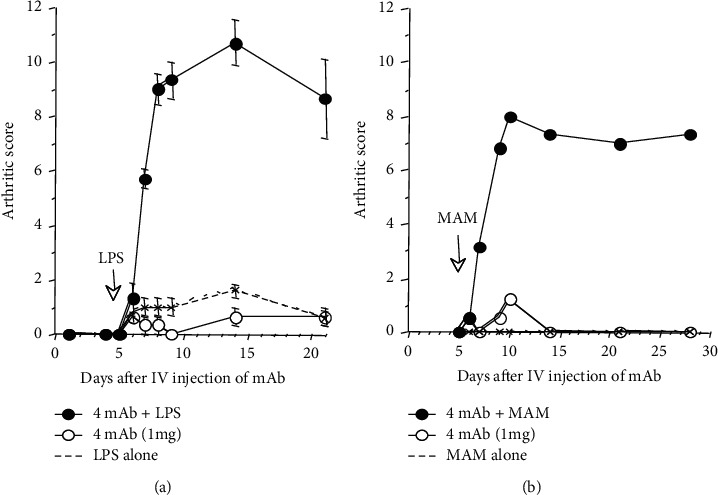
Synergistic effects of bacterial toxins to trigger and exacerbate arthritis in CAIA mice. CAIA in two mouse strains (DBA/1 and B10.RIII) was established by IV injection of the arthritogenic four-clone monoclonal antibody cocktail (1 mg) on day 0 and subsequent IP injection of 50 *μ*g of lipopolysaccharide (LPS) from *E. coli* (a), or mycoplasma arthritidis mitogen (MAM) (b) on day 3. Mice developed arthritis within 24–48 hours after receiving the toxins and the arthritic score plateaus 7-8 days later and can last up to 30 days. (a) mAb plus LPS in DBA/1 mice. (b) mAb plus MAM in B10.RIII mice.

CIA severity was enhanced by oral (PO) administration of *mycoplasma arthritidis* mitogen (MAM) [44], a T cell mitogen, or *Staphylococcal* enterotoxin B from *Staphylococcus aureus* (SEB) [45] before and after arthritis onset. In CAIA, we studied the arthritogenic effect of lipopolysaccharide derived from *Escherichia coli O111:B4* (*E. coli*-LPS), a bacterial toxin produced from intestinal Gram-negative bacteria. Although the IV administration of a low dose (1 mg) of the four-clone monoclonal antibody cocktail failed to induce arthritis, a subsequent intraperitoneal (IP) administration of *E. coli*-LPS (50 *μ*g) on day 3 induced severe arthritis within 24–48 hours, which reached a plateau after 7-8 days (Figure 4(a)).

While neither the low-dose antibody cocktail or LPS alone was able to induce arthritis, the combination induces arthritis synergistically. Similar synergistic effects with the antibody cocktail were observed with MAM) (Figure 4(b)) and SEB (data not shown). This indicated that bacterial toxins nonspecifically play critical pathogenic roles with autoantibodies and contribute to the induction and progression of arthritis.

### 2.3. Attentions in CIA and CAIA as Models of RA

#### 2.3.1. Differences between CIA and CAIA

The susceptibility of CIA and CAIA depends on the MHC H-2 haplotypes and species of heterogeneous type II collagen used for immunization. In mouse CIA, H-2q (DBA/1), H-2r (B10.RIII) and H-2s (SJL) haplotypes show high CIA susceptibility [24, 46, 47], while H-2b (C57BL/6) shows low CIA susceptibility, as observed in their respective levels of serum anti-mouse type II collagen autoantibodies. To induce CIA in these low responder strains, changing the immunological methods, such as an extra booster injection of heterologous type II collagen emulsified 1 : 1 with CFA containing a high concentration (5 mg/ml) of *Mycobacterium tuberculosis (M. tuberculosis)* on day 21 [48, 49], or the inhibition of cytokines such as IFN-*γ* [50], induce more frequent arthritis, and are useful for a specific experimental purpose. For gene function analysis, establishing gene modified mice requires 1-2 years or more. As C57BL/6 mice, which are a common transgenic mouse strain, are low responders to CIA induction, they may need to be backcrossed with highly susceptible DBA/1 mice [48]. In some cases, reproducing the arthritic phenotype in neonate mice is unreliable and inconsistent.

On the other hand, because CAIA bypasses anti-type II collagen autoantibody production, CAIA can be induced at 100% incidence, in a shorter period, and in many mouse strains who carry H-2q (DBA/1), H-2r (B10.RIII), H-2b (C57BL/6), H-2d (BALB/c) [42], and H-2s (Scid/scid C.B-17) [43]. An IP injection combining the monoclonal antibody cocktail and LPS reproducibly induces especially severe arthritis in widely available strains such as BALB/c, as well as C57BL/6 mice which is available for genetic modifications. Therefore, CAIA is a more useful model for the functional analysis of arthritis-related genes using various congenic, transgenic, and knockout mice [51].

Similar to human RA, CIA mice show increased rheumatoid factor (RF) and anti-citrullinated protein/peptide antibodies (ACPA) [52, 53]. Although humans tend to show a sex-bias in RA development [54, 55], mice did not seem to show any sex-bias in CIA [55] and CAIA (unpublished data). Interestingly, the sex-bias could be reproduced in human gene transgenic mice. With inducing human HLA-DR4 genes in mice, the female mice developed higher incidences of severe arthritis than male mice [56]. Thus, the transgenic mice can be useful for analyzing human genes related to RA development in CIA.

Table 1 shows the similarities and differences between CIA and CAIA models and compares these with the features of human RA.

#### 2.3.2. Incidence and Severity of CIA: Study Variations by Emulsion and Animal Housing

Although CIA is a useful model to study the mechanism of arthritis induction, some important points should be considered to reduce variability in CIA. It is very difficult to obtain consistent reproducibility among long-term animal experiments. CIA would be considered a long-term experiment as it requires four weeks for developing arthritis and another three weeks to reach maximum severities of arthritis. For example, the incidence and severity of arthritis can range from 0% to 100%, just depending on the cage.

Housing conditions can affect the phenotype of CIA. Animals must be housed in specific pathogen-free (SPF) conditions without stress. Due to the aggressive nature of select mouse strains (DBA/1), fighting among cage-mates may be inevitable, leading to injuries and stress which may affect CIA development.

Qualities of the emulsion of CFA and type II collagen used to immunize mice are also important. When inducing CIA in many mice, a higher volume of emulsion is required and may need to be prepared with several emulsion batches. The qualities and stabilities of the emulsions in between the preparations can affect the consistency of developing CIA.

To obtain better results with these variations, the same group of mice should not be placed in a single cage; instead, the group of mice should be housed randomly in several cages. However, CAIA can avoid these problems because it yields approximately 100% incidence with reproducible arthritis severity in short periods (within 2 weeks).

#### 2.3.3. Importance of a Negative Control Group: Immunological Effects of CFA and LPS in Arthritis Induction

In immunological function research, the most critical consideration in CIA is the effect of CFA which affects immune cells and the intestinal barrier [57, 58]. CIA requires subcutaneous (SC) injection of an emulsion of CFA with type II collagen into mouse tails. Therefore, a negative control group consisting of mice injected with an emulsion of CFA with PBS should be included to assess the effects of CFA in host immune systems. T cells in CIA mice are predominantly activated by the CFA in the emulsion, not by type II collagen. Dynamics of inflammatory or anti-inflammatory cytokines and chemokines in published reports might, in reality, be related to T cells affected by an adjuvant, such as CFA [59, 60]. With the same concept in mind for CAIA experiments, a negative control group consisting of an LPS injection alone should be used.

## 3. Risk Factors in Pathogenesis of RA

### 3.1. Internal Factors for RA

RA is a complex inflammatory disease associated with many inflammatory factors, such as prostaglandins, cyclooxygenase I and II, complement system, inflammatory and anti-inflammatory cytokines, chemokines, and signal transduction of immune cells, which are closely related to the enhancement and repression of inflammatory reactions. Therefore, these factors have been used as target molecules for reducing inflammation by anti-RA drugs including inhibitors, antagonists, and antibodies. On the other hand, the contribution of specific genes in arthritis induction has been also determined using genetic modification technologies such as congenic, transgenic, and knockout mice. Many researchers have been studying the role of T cells in the production of various cytokines and chemokines in inflammation, and the roles of transcriptional factors associated with the production of the internal factors [61].

However, these inflammatory factors in RA might be affected not only by the internal immune responses but also by external pathological factors. Identifying external environmental factors which affect the inflammatory responses must be the next essential step to understand pathogenesis in RA.

### 3.2. Dysbiosis as an External Factor in RA: A Balance between Intestinal Bacteria, Mucosal Barrier, and Immune Function of the Digestive Tract

Generally, in the digestive tract, physiological inflammation (homeostasis) is maintained by balancing the mucosal immune function and barrier function, and intestinal bacterial composition. However, this balance may be disrupted by increased intestinal mucosa permeabilities [19, 62] which are affected by drugs, such as nonsteroidal anti-inflammatory drugs(NSAIDs), intestinal disorders caused by diarrhea and constipation [63], and mental stress [64–66]. As a result, excessive environmental factors absorbed from the intestinal mucosa shift physiological inflammation to pathological inflammation, leading to chronic inflammatory diseases. Indeed, sera and synovial tissues from RA patients contained bacterial DNA and peptidoglycan [67], suggesting the dysfunction of their mucosal barriers.

Recent reports suggested that vegetarian diets can change the fecal bacterial population in RA patients, subsequently causing remission of clinical symptoms [68, 69]. These results may indicate a relationship between intestinal bacteria and RA pathology [14–18]. More specifically, 16S ribosomal RNA analysis, which can analyze fecal bacteria, demonstrated existing an imbalance of intestinal bacteria in RA patients. These results suggest that the dysbiosis of intestinal commensal bacteria, rather than specific individual bacteria, is related to the pathogenesis of RA. Moreover, as dysbiosis was also observed in inflammatory bowel disease (IBD) [16, 17] and, in spondyloarthritis [70], it might be a common condition in many autoimmune diseases.

For example, it was reported that *Bifidobacteria, Bacteroides-Porphyromonas-Prevotella* group, *Bacteroides fragilis* subgroup, and *Eubacterium rectale-Clostridium coccoides* group microbes were significantly decreased in RA patients compared with fibromyalgia patients [14]. Moreover, it was also reported that *Prevotella copri* levels increased and *Bacteroides* levels decreased in the feces of new-onset untreated RA patients [71]. An analysis of bacteria in fecal, dental, and saliva samples by metagenomic shotgun sequencing and a metagenome-wide association study compared RA patients with healthy volunteers. The study revealed that no *Haemophilus* but increased *Lactobacillus salivarius* in RA patients [72].

## 4. Examples of RA Risk Factor Studies Using CIA and CAIA

As described above, CIA and CAIA are very useful models to identify RA-related factors and to study the roles of these factors in the pathogenesis of arthritis. Here, we introduce some suggested experiments using modified protocols based on CIA and CAIA as follows.

### 4.1. Oral Collagen-Induced Arthritis (Oral CIA): A Model to Mimic Intestinal Absorption of Antigens

**Figure 5:**
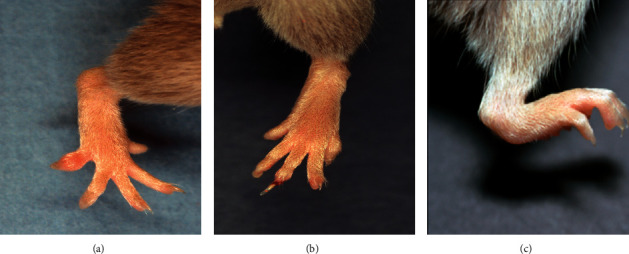
Oral Collagen-Induced Arthritis (oral CIA) and nonantibody mediated LPS-induced arthritis. Oral administration of heterologous type II collagen for a prolonged period can elicit very mild arthritis (oral CIA) in mice, (a) and (b), compared with typical severe arthritis in general CIA without LPS injection (c).

DBA/1 mice received oral administration of heterologous type II collagen for 15 weeks (5 times/week) and developed low severity arthritis as shown in Figures 5(a) and 5(b) (Oral CIA), accompanied by increased anti-MsII antibodies. This study indicated that ingesting dietary heterologous type II collagen for many weeks developed low levels of antibodies against MsII, which can induce arthritis [73].

### 4.2. Nonantibody Mediated LPS-Induced Arthritis: Induction by LPS Alone in Immune-Deficient Mice

In continuation of the previous section above, oral coadministration of LPS (10 *μ*g) in the Oral CIA mice led to more severe arthritis associated with higher antibody levels against MsII. To confirm the role of intestinal permeability in this synergistic effect of LPS, the mice orally received a normal dose of indomethacin (40 *μ*g/mouse) and ovoinhibitor (2 mg/mouse), a protease inhibitor in egg white, known to enhance the permeability of the digestive tract. These mice showed suppressed immune function of the digestive tract, along with low levels of antibodies against the orally administered type II collagen. On the other hand, these mice showed higher susceptibility against LPS, resulting in 50% mortality of mice after the IP injection of 50 *μ*g LPS. In addition, the surviving mice developed very severe arthritis without antibodies against MsII. This kind of severe arthritis had not been observed in general CIA and CAIA (Figure 5(c)) [73]. This mouse arthritis model may mimic pathologically similar arthritis in RA patients without HLA-DR4 gene and autoantibodies. These results suggest the importance of mucosal barrier permeability and absorption of bacterial toxins, such as LPS, in the digestive tract in disease induction.

### 4.3. Studies of the Effect of Bacterial Toxins in CAIA

As described before, an IP injection of bacterial toxins such as LPS, MAM, and SEB after an initial IV injection of a low dose of anti-typeII collagen antibody cocktail (1 mg) induced severe arthritis (Figure 4), indicating the model can be used for studying the effects of environmental factors in arthritis induction. Therefore, pathological effects of LPS from *Porphyromonas gingivalis* (*P. gingivalis)* (*Pg*-LPS), which cause periodontitis and contribute to progressive RA [74–76], were studied using this protocol. An IP injection of *Pg*-LPS (50 *μ*g) failed to develop arthritis in DBA/1 mice who initially received an IV injection of the type II collagen antibody cocktail (1 mg) (data not shown) although *E. coli*-LPS (50 *μ*g) induced severe arthritis under the same conditions. These results suggest the pathological role of *P. gingivalis* in inducing arthritis may be unrelated to Pg-LPS, but rather associated with changing the intestinal bacteria composition and mucosal barrier function, as reported by Nakajima et al. [77]. Inducing arthritis using a combination of the anti-type II collagen antibody cocktail and bacterial toxins might be useful to screen environmental factors and study their pathological roles in arthritis induction.

### 4.4. Studies of the Pathological Roles of Intestinal Bacteria Toxins in CAIA: Influence of Aging

Yoshino et al. reported that the oral administration of LPS on day 50 after type II collagen immunization rebooted severe arthritis in DBA/1 mice in the CIA model [78]. This LPS administration was tested in the CAIA model using mice aged 8 weeks and 8 months. In the 8-month-old mice, 1 mg of LPS orally given on day 3 after the type II collagen antibody cocktail injection (1.5 mg) induced arthritis within 24–48 hours and reached a plateau after 7-8 days with high serum IL-6 levels. The severity of arthritis correlated with serum IL-6 levels. However, in the 8-week-old mice,this same procedure failed to develop arthritis and also to increase serum IL-6 levels. This result indicated that increased animal age is related to lowered intestinal barrier function and increased susceptibility against LPS toxicity due to a decreased mucosal defense function [79]. These protocols might be useful to evaluate increased permeability and decreased barrier function and their correlation with sensitivity against bacterial toxins related to aging and intestinal diseases.

### 4.5. Studies of Intestinal Bacterial Effects on Arthritis

Bacterial species in the oral cavity and intestine may be linked to MHC molecules in humans and mice [80]. Totaro et al. showed that DNA from *P. gingivalis*, which is a pathogenic bacteria strain, was found at a higher rate in the synovial membranes of RA patients who have the HLA-DR*β*1 allele than in other patients [81]. In addition, Gomez et al. showed that *Clostridium*-like bacteria are dominant in the intestine of CIA-sensitive DR*β*1 0401 allele transgenic mice, while *Porphyromonadaceae* and *Bifidobacteria* are dominant in CIA-resistant DR*β*1 0402 allele transgenic mice. The DR*β*1 0402 mice also showed sex and age-influenced gut microbiome composition, but not the DR*β*1 0401 mice who even had altered gut permeability [82]. Recently, several reports suggested a link between gut bacteria and CIA development using oral administration of several bacteria strains in mice. For example, among several beneficial bacteria strains altering disease outcomes [83], *Lactobacillus helveticus* reduced the severity of arthritis significantly [84]; *Lactobacillus casei* administration during induction of CIA suppressed anti-type II collagen antibody levels and delayed onset and reduced severity of CIA [85]. Interestingly, not only live bacteria but also heat-killed *Lactobacillus reuteri* administration inhibited CIA and CAIA induction [86]. Moreover, Liu et al. compared fecal bacteria species between arthritic and nonarthritic DBA/1 mice in CIA and demonstrated *Lactobacillus* was dominant in arthritic mice but,*Bacteroidaceae* and *Lachnospitaceae* proliferated in nonarthritic mice. They also showed that germ-free mice can become CIA-susceptible when they receive intestinal bacteria from CIA arthritic mice [57].

Alternatively, Jubair et al. reported effects of mixed broad-spectrum antibiotics (ampicillin, metronidazole, neomycin, and vancomycin), which change intestinal bacterial composition, in CIA [58]. Eliminating intestinal bacteria with the antibiotic treatments suppressed the severity of arthritis by approximately 40% and decreased anti-type II collagen antibody levels. Interestingly, dysbiosis and inflammation of the intestinal mucosa had already occurred before the onset of arthritis in DBA/1 mice who were immunized with an emulsion of CFA and type II collagen. These mice also have higher intestinal mucosa permeability confirmed by oral administration of fluorescein isothiocyanate (FITC) conjugated dextran. Another study showed that the administration of mixed broad-spectrum antibiotics reduced severity of arthritis, correlating with decreased Th17 cells and their IL-17 production in the intestinal lamina propria of the mice [87, 88].

**Figure 6:**
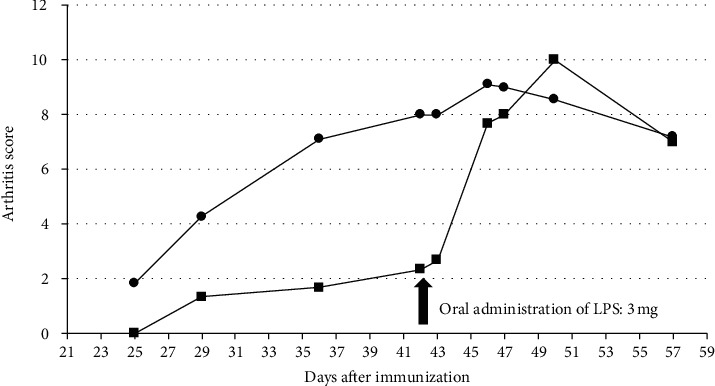
Exacerbating arthritis by oral administration of LPS in nonarthritic CIA mice. Mice were immunized with an emulsion of type II collagen and 1 mg/ml CFA. Nonarthritic mice failed to develop significant arthritis; however, the oral administration of LPS on day 42 induced severe arthritis in the nonarthritic mice (square), similar to the arthritic mice (circle).

These findings suggested CFA may affect bacteria composition, host immune systems, and barrier functions of the intestinal mucosa in CIA. To confirm this hypothesis, 8-week-old DBA/1 mice receiveda single immunization with an emulsion of type II collagen and CFA containing low concentration of *M. tuberculosis* (1 mg/ml). This immunization generally fails to induce CIA. Six weeks after the initial immunization, an oral administration of LPS (3 mg) induced arthritis in nonarthritic mice (Figure 6). These results indicate that the 14-week-old mice, who are usually resistant to LPS by oral administration, absorbed LPS from the intestine. This result indicated that CFA significantly affects mucosal barrier functions and increases the absorption of bacterial toxins, such as LPS, which are generally not absorbed in intestine.

These results clearly showed that dysbiosis, the bacterial composition change in the intestine, is closely related to the induction, progression, and severity of arthritis. These results might adapt to studies using gnotobiotic mice with which intestinal bacteria are removed or transferred. Furthermore, these studies suggested that CFA affects host immune systems and intestinal bacteria, resulting in inflammation of the intestinal mucosa. Thus, CFA plays important role in the development of arthritis, which is useful knowledge for future studies.

### 4.6. Studies on the Effects of Dysbiosis and Oral Bacteria on Arthritis

Dental clinical research reported that RA patients with periodontitis, especially associated with *P. gingivalis* [89] and *Aggregatibacter actinomycetemcomitans* [90], suffer from more severe arthritis [75–77]. It was demonstrated that *P. gingivalis* infections, which led to periodontitis, aggravated arthritis in CIA mice [91–95]. Regarding pathological roles of *P. gingivalis*, Nakajima et al. recently published that oral administration of *P. gingivalis* in C57BL/6 mice changed bacterial flora composition. The change suppressed mRNA expression of tight junction proteins in mucosal membranes and induced high serum endotoxin levels by increasing the permeability of the intestinal mucosal barrier [77]. The same group reported that the repeated oral administration of *P. gingivalis* exacerbated CIA, with increased numbers of intestinal bacteria, such as *Bacteroides* and *Prevotella*,leading to induce the proliferation of Th17 cells and increase blood IL-17 levels [96]. However, the oral administration of *P. gingivalis* did not change the anti-type II collagen antibody levels and failed to activate*P. gingivalis* proliferation in the intestine. Dysbiosis can also be developed by oral antibiotics. Depleting the microbiota with oral feeding of an antibiotic cocktail prior to the induction of CIA reduced severity of arthritis and the levels of serum inflammatory cytokines and anti-CII antibodies [58]. However, enrofloxacin, an antibiotic, aggravated CIA and increased the levels of serum IFN-g, IL-17A, and IL-6 [97].

Furthermore, it is important to note the intestinal bacteria composition changes during the development of CIA [57, 87]; *Firmicutes* and *Proteobacteria* were increased and *Bacteroides and lactobacillus* were decreased. Even progressing arthritis development shifts bacteria composition in CIA mice [98]. The composition change may be associated with the stress and pain in the developing disease as well as housing conditions [57, 58, 97]. The housing condition factor requires more attention because viral or bacterial contamination in mice can reduce the severity of arthritis in CIA [99] and CAIA (unpublished observed data). Therefore, specific pathogen-free conditions are highly recommended in the arthritis studies, at least.

Further studies are required to analyze pathogenesis of dysbiosis in diseases. CIA and CAIA are useful models because oral inoculation of a target bacterium in mice is an easy procedure and then observe the dysbiosis and the resulting impact on the severity of arthritis.

### 4.7. Studies of the Adverse Effects of Antiautoimmune Drugs that Influence the Intestinal Bacteria

The administration of immunosuppressants, steroids, and NSAIDs, which are used to treat autoimmune diseases, may cause intestinal dysbiosis. Among these drugs, methotrexate (MTX) has been used as an effective positive control drug in both CIA and CAIA [100, 101]. However, it was reported that MTX treatment decreased the numbers of *Prevotella* [102], *Enterococcus faecium* [103], and *Bacteroides group* [104] in the intestines of RA patients. Bacteria number changes in the intestinal flora including these bacteria might be closely related to the effectiveness of MTX and remission of RA. Our report indicated that the IgA and IgG levels against *P. gingivalis* significantly increased in sera from RA patients treated with MTX for a long period, suggesting MTX may change the intestinal bacterial balance including *P. gingivalis* [20, 100, 105]. As previously described, immune suppressed mice become highly susceptible against LPS toxicity and develop severe and destructive arthritis with a single IP injection of LPS. Therefore, a long duration of treatment with immunosuppressive RA drugs might further reduce the immune function in RA patients, who have already a low immune response and worsen their RA prognosis.

Recently, it has been reported that immune checkpoint inhibitors, such as programmed cell death-1 (PD-1) and programmed death-ligand 1 (PD-L1) activate T cells and can induce autoimmune-disease-like symptoms [106–108]. However, in CIA using DBA/1 mice, the administration of anti-PD-L1 antibodies decreased the severity of arthritis and serum IL-17 and IL-23 levels [109]. On the other hand, administration of soluble PD-1, which was detected at high levels in the sera or synovial fluid in RA patients, aggravates CIA by activating Th1 and Th17 cells [110]. In RA patients, the functional balance between Th17 cells and Tregs was changed [111].Moreover, in CIA, the administration of IL-27, which suppresses Th17 differentiation, decreased the severity of CIA and increased Th17 positive Tregs in the spleen [112].

Activating T cells by suppressing the PD-1/PD-L1 pathway with immune checkpoint inhibitors induces autoimmune-disease-like-symptoms in cancer patients, but it is still under investigation as to how the activation of pathways of Th17 or Tregs affects the phenotypes in CIA and CAIA. In addition, the relationship between the immune checkpoint inhibitors and intestinal bacteria should be closely analyzed. CIA and CAIA might be useful models to investigate the relationships between cancer immunity and the induction of autoimmune-disease-like symptoms.

## 5. Conclusion

Recently, many studies have been reported on the relationship between autoimmune diseases and intestinal bacteria. Many autoimmune diseases including RA,which are considered chronic diseases, have common pathogenesis in decreasing intestinal barrier function and mucosal immune function. CIA and CAIA, animal models of arthritis, have been used for many inflammation related studies, but not sufficiently used for studies investigating bacterial flora in the pathogenesis of arthritis. 

As described in this review, CIA and CAIA can be used for studying the contribution of gene background, host immune ability, bacterial flora, and intestinal mucosal barriers to the pathogenesis of RA, using many parameters beyond just arthritic scores. We believe that the CIA and CAIA models and their published protocols will be useful to investigate the many factors involved in autoimmune diseases, and we hope this review proves to be a useful guide for studying new concepts in many diseases.

## Figures and Tables

**Table 1:** Similarities and differences between CAIA and CIA animal models. CFA: Complete Freund's adjuvant, RA: rheumatoid arthritis, CIA: Collagen-Induced Arthritis, and CAIA: Collagen Antibody-Induced Arthritis.

	CIA model	CAIA model
Induction method	Type II collagen/CFA	Monoclonal antibodies against type II collagen
Feature of arthritis	Polyarthritis	Polyarthritis
Similarities to human RA	(i) Activates adaptive immune system. (ii) Produces antibodies against specific epitopes on type II collagen in joints. (iii) Synovial inflammation due to infiltrating immune cells. (iv) Cartilage and bone destruction. (v) Increases RF and ACPA	(i) Antibodies against specific epitopes on type II collagen in joints. (ii) Synovial inflammation due to infiltrating immune cells. (iii) Cartilage and bone destruction.
Advantages	(i) Study CIA development in relation to the adaptive immune systems. (ii) Study immunity specific to cartilage. (iii) No sex-bias	(i) Induces arthritis in many mouse strains. (ii) Develops consistent arthritis severity and with almost 100% incidence. (iii) Effectively studies test articles in a short study period (1-2 weeks). (iv) RF and ACPA negative. (v) No sex-bias
Disadvantages	(i) Restricted to select mouse strains. (ii) Animal housing conditions can affect arthritis severities and incidences. (iii) A long study period due to the slow onset of arthritis (1-2 months). (iv) Strongly affected by CFA presence.	(i) Does not involve the full spectrum of immune activation. (ii) Animal housing conditions can affect arthritis severities and incidences.
